# Annexin Peptide Ac2-26 Suppresses TNFα-Induced Inflammatory Responses via Inhibition of Rac1-Dependent NADPH Oxidase in Human Endothelial Cells

**DOI:** 10.1371/journal.pone.0060790

**Published:** 2013-04-24

**Authors:** Hitesh M. Peshavariya, Caroline J. Taylor, Celeste Goh, Guei-Sheung Liu, Fan Jiang, Elsa C. Chan, Gregory J. Dusting

**Affiliations:** 1 O’Brien Institute, University of Melbourne, Fitzroy, Victoria, Australia; 2 Centre for Eye Research Australia, University of Melbourne, East Melbourne, Victoria, Australia; 3 Faculty of Health Sciences, The Australian Catholic University, Victoria, Australia; 4 Key Laboratory of Cardiovascular Remodeling and Function Research, Chinese Ministry of Education and Chinese Ministry of Health, Qilu Hospital, Shandong University, Jinan, Shandong Province, China; Sun Yat-sen University Cancer Center, China

## Abstract

The anti-inflammatory peptide annexin-1 binds to formyl peptide receptors (FPR) but little is known about its mechanism of action in the vasculature. Here we investigate the effect of annexin peptide Ac2-26 on NADPH oxidase activity induced by tumour necrosis factor alpha (TNFα) in human endothelial cells. Superoxide release and intracellular reactive oxygen species (ROS) production from NADPH oxidase was measured with lucigenin-enhanced chemiluminescence and 2′,7′-dichlorodihydrofluorescein diacetate, respectively. Expression of NADPH oxidase subunits and intracellular cell adhesion molecule (ICAM-1) and vascular cell adhesion molecule (VCAM-1) were determined by real-time PCR and Western blot analysis. Promoter activity of nuclear factor kappa B (NFκB) was measured by luciferase activity assay. TNFα stimulated NADPH-dependent superoxide release, total ROS formation and expression of ICAM-1and VCAM-1. Pre-treatment with N-terminal peptide of annexin-1 (Ac2-26, 0.5–1.5 µM) reduced all these effects, and the inhibition was blocked by the FPRL-1 antagonist WRW4. Furthermore, TNFα-induced NFκB promoter activity was attenuated by both Ac2-26 and NADPH oxidase inhibitor diphenyliodonium (DPI). Surprisingly, Nox4 gene expression was reduced by TNFα whilst expression of Nox2, p22phox and p67phox remained unchanged. Inhibition of NADPH oxidase activity by either dominant negative Rac1 (N17Rac1) or DPI significantly attenuated TNFα-induced ICAM-1and VCAM-1 expression. Ac2-26 failed to suppress further TNFα-induced expression of ICAM-1 and VCAM-1 in N17Rac1-transfected cells. Thus, Ac2-26 peptide inhibits TNFα-activated, Rac1-dependent NADPH oxidase derived ROS formation, attenuates NFκB pathways and ICAM-1 and VCAM-1 expression in endothelial cells. This suggests that Ac2-26 peptide blocks NADPH oxidase activity and has anti-inflammatory properties in the vasculature which contributes to modulate in reperfusion injury inflammation and vascular disease.

## Introduction

Annexin-1 (also termed lipocortin-1) is the first member of the annexin superfamily which, in humans, consists of at least 12 members each of which has a unique N-terminal sequence [1]. The annexin-1 peptide and its N-terminal derivative Ac2-26 have been shown to have protective effects in both brain and cardiac tissue following ischemia/reperfusion injury [2], [3], [4]. These effects have been attributed to the anti-inflammatory actions of annexin-1 and Ac2-26.

Annexin-1 is found intracellularly in gelatinase granules of neutrophils [5] and in human serum, particularly in inflammatory conditions such as myocardial infarction [6] and colitis [7]. These findings, together with observations from *in vitro* and *in vivo* models of inflammation strongly suggest an anti-inflammatory role for annexin-1. When released, annexin-1 binds to its receptor to mediate cell detachment and inhibits leukocyte transmigration [5], [8], [9]. Data now suggest that these effects are mediated through the specific interaction of annexin-1 with the formyl peptide receptor (FPR) [10] and FPR like receptor (FPRL-1) [11].

During an inflammatory response, endothelial cells become activated, a process characterized by specific changes in endothelial phenotype including upregulation of cell surface adhesion molecules which enhance leukocyte adhesion and transmigration across the blood vessel wall (reviewed by [12]). There are a number of stimuli for endothelial activation including inflammatory cytokines (e.g. Interleukin-1β, tumour necrosis factor α; TNFα), ischemia-reperfusion and diabetes [13], [14], [15]. Reactive oxygen species (ROS) are known to be involved in many of the processes involved in endothelial activation including upregulation of adhesion molecules such as intracellular cell adhesion molecule (ICAM-1), vascular cell adhesion molecule (VCAM-1), monocyte chemoattractant protein-1 (MCP-1) and P-selectin [16], [17], [18]. For example, TNFα-induced intracellular ROS regulates downstream NFκB signaling pathways and the expression of ICAM-1, VCAM-1 and MCP-1 in endothelial cells [16], [19], [20].

The potential sources of ROS in endothelial cells are multiple including the mitochondrial electron transport chain, NADPH oxidase, xanthine oxidase, cytochrome P450 and uncoupled endothelial NO synthase (eNOS). Of these, NADPH oxidase is the only enzyme which is dedicated to ROS production [15], [21]. There are seven isoforms of the catalytic subunit (Nox1 to Nox5, Duox1 and 2) of NADPH oxidase, of these Nox2, Nox4 and Nox5 isoforms are expressed in endothelial cells [22], [23], [24], [25]. The proinflammatory cytokine TNFα has been shown to activate acutely NADPH oxidase assembly via a protein kinase C (PKC) dependent pathway [2], [17]. Furthermore, TNFα-mediated inflammatory effects such as increased expression of cell adhesion molecules (ICAM-1 and VCAM-1) have also been attributed to the activation of NADPH oxidase [16], [26]. Intervention of TNFα-induced Rac1-depedendent NADPH oxidase assembly either by dominant negative Rac1 (competitive inhibitor of Rac1) or cells derived from p47phox knock out animal significantly reduced ICAM-1 and VCAM-1 expression in endothelial cells [2], [17], [19], [27]. These findings suggest TNFα enhanced NADPH oxidase activity and expression of cell adhesion molecules in endothelial cells.

Whilst the anti-inflammatory and anti-migratory effect of annexin-1 on leukocytes is well established, little information is available regarding the biological effect of this peptide on endothelial cells during inflammation. In this study, we investigated the effects of the annexin-1 peptide Ac2-26 on TNFα-induced NADPH oxidase-derived ROS production and its role in endothelial cells during inflammation. To the best of our knowledge this is the first study to show that annexin-1 peptide Ac2-26 inhibits NADPH oxidase activity and the inflammatory response in endothelial cells.

## Materials and Methods

### Reagents

The following reagents used in the study were obtained from Sigma-Aldrich Australia (Castle Hill, New South Wales, Australia): allopurinol, diethyldithiocarbamic acid, diphenyleneiodonium (DPI), lucigenin, NADPH (reduced β-nicotinamide adenine dinucleotide phosphate), indomethacin, N-nitro-L-arginine (L-NAME), rotenone and TNF-α. Ac2-26 was obtained from Pheonix Pharmaceuticals Inc. (Burlingame, CA, USA). FPRL-1 antagonist WRW4 (Trp-Arg-Trp-Trp-Trp-Trp-NH_2_) was obtained from Tocris Bioscience (Bristol, UK).

### Cell Culture

Human dermal microvascular endothelial cells (HMECs) kindly provided by Prof. Thomas J Lawley from Centers for Disease Control and Prevention, Atlanta, GA, USA. Dermal microvascular endothelial cells initially obtained from human foreskin and immortalised with a PBR-322-based plasmid containing the coding region for the simian virus 40A gene product, large T-antigen [28], were cultured in EGM-MV Bulletkit (Lonza) containing 10% fetal bovine serum in endothelial basal medium using standard cell culture techniques.

### Measurement of Superoxide

NADPH oxidase activity was assessed by measuring superoxide with lucigenin-enhanced chemiluminescence using a Topcount microplate scintillation counter (PerkinElmer, Waltham, Massachusetts, USA) running in single-photon-count mode, as described previously [25]. For HMECs, (10,000 cells/well) were cultured in white OptiPlates (PerkinElmer) for 24 hr. Media was replaced at this time and cells were treated with TNF-α (20 ng/ml) for various times. Inhibitors were added 30 min prior to application of TNF-α. Before measurement of superoxide, cells were preincubated with diethyldithiocarbamic acid (3 mM) in Krebs-HEPES buffer (HBSS, in mM: NaCl 98.0, KCl 4.7, NaHCO_3_ 25.0, MgSO_4_ 1.2, KH_2_PO_4_ 1.2, CaCl_2_ 2.5, D-glucose 11.1 and Hepes-Na 20.0) for 45 min to inactivate endogenous superoxide dismutase. The endothelial NADPH oxidase was stimulated with 100 µM NADPH, and the chemiluminescence was detected with 5 µM lucigenin.

### Measurement of Intracellular ROS

Total intracellular ROS measurement in endothelial cells was also performed using 2′,7′-dichlorodihydrofluorescein diacetate (DCFH_2_-DA; Invitrogen, Life Technologies, Victoria, Australia) fluorescence as previously reported [25]. HMECs cultured in 96 well (black) opti-plates (10,000 cells/well) were washed with HBSS prior to loading with DCFH_2_-DA (10 µM). Fluorescence was then measured with excitation and emission wavelengths of 480 nm and 520 nm respectively using a Polarstar microplate reader (BMG Labtech, Germany) at 37°C over a period of 1 hr.

### MTS Assay

A tetrazolium-based proliferation assay was used to determine HMEC cell number following superoxide or ROS measurement. Cells were washed with PBS and incubated with 10 µl of CellTiter 96™ AQ solution (Promega, New South Wales, Australia) and 40 µl of Krebs-HEPES buffer for 1 h at 37°C in a 5% CO_2_ incubator. Absorbance at 490 nm was determined using a Polarstar plate reader after incubation. Luminescence count (Count per second; CPS) or fluorescence unit (Relative fluorescence unit; RFU) were normalized with MTS absorbance and value expressed as percentage control.

### Transfection of Plasmids

HMECs (100, 000 cells/well) were seeded in six-well plate the day before transfection. Transfection was performed using Lipofectamine2000. Each well contained 500 ng of either green fluorescent protein (GFP) or N17Rac1 (kindly provided by Prof. Richard G Pestell Kimmel Cancer Center, Thomas Jefferson University Philadelphia, PA 19107) mixed with 1 µL of Lipofectamine2000 in the presence of 500 µl of Opti-MEM. Cells were transfected for 5 h and then incubated with complete medium for 48 h. Transfection efficiency was determined by counting GFP positive cells vs. total cells. We were able to achieve 70 to 80% GFP positive cells (data not shown). The transfected cells were then treated with TNFα in the absence or presence of Ac2-26 peptide or DPI for 6 h.

### Real-time PCR

HMECs (100,000 cells) were collected in 0.5 ml TriReagent (Ambion, Austin, TX, USA). The total RNA was extracted according to the manufacturer's protocol. Total RNA was reverse-transcribed to cDNA (100 ng) using random hexamers and high capacity TaqMan reverse transcription reagents (Applied Biosystems, Foster City, CA, USA) at 37°C for 2 h followed by 95°C for 10 min. Real-time PCR reactions were performed in a 7300 real-time PCR system (Applied Biosystems) using TaqMan Universal PCR master mix and predesigned gene-specific probe and primer sets for ICAM-1 (Hs00277001_m1), VCAM-1 (Hs00365486_m1), Nox4 (Hs01558199_m1), p22phox (Hs00164370_m1) and p67phox (Hs00166416_m1; TaqMan Gene Expression Assays, Applied Biosystems). Human GAPDH (4326317E) was used as the housekeeping gene.

### Gene Specific Primer PCR

Due to the inconsistency of Nox2 gene expression detection with Applied Biosystems TaqMan Assay on demand, we used gene specific amplification of cDNA for detection of Nox2 gene expression. cDNA was prepared from 200 ng of total RNA. cDNA was generated with the Thermoscript RT-PCR System (Invitrogen, Carlsbad, CA) using gene-specific priming with Nox2-For AGAGGGTTGGAGGTGGAGAATT (Accession No. NM_000397) and GAPDH- For GAAGGTGAAGGTCGGAGTC (Accession No. NM_002046) at an annealing/extension temperature of 55°C. Human GAPDH was used as a housekeeping gene to normalise all samples. The real-time PCR reactions were performed in a 7300 real-time PCR system (Applied Biosystems) using SYBR Green-based real-time PCR assay with the SYBR Green PCR Master Mix (Applied Biosystems) and in-house designed primers against Nox2 (For AGAGGGTTGGAGGTGGAGAATT and Rev, GCACAAGGAGCAGGACTAGATGA; Accession No. NM_000397) and GAPDH (For GAAGGTGAAGGTCGGAGTC and Rev, GAAGATGGTGATGGGATTTC; Accession No. NM_002046). The specificity of the products was demonstrated by melt curve analysis and gel electrophoresis.

### Western Blot Analysis

Cells (500, 000 cells/dish ) cultured in 100 mm dishes were washed with cold PBS and lysed with 100 µl of cell lysis buffer (pH 7.5) containing 100 mM NaCl, 1% Triton X-100, 10 mM Tris, 2 mM EDTA and a protease inhibitor cocktail (Roche). Cell lysates were centrifuged at 13,000 rpm at 4°C for 15 min and the supernatants were mixed with (6X) Laemmli buffer and boiled for 5 min. Equal amounts of protein were separated by 10% SDS-PAGE and transferred to Hybond nitrocellulose membranes (Amersham). The membrane was blocked with 5% non-fat milk powder in TBS (pH 7.5) and hybridized overnight at 4°C with primary antibody ICAM-1 (Research Diagnostics Inc, 1∶200) VCAM-1 (Research Diagnostics Inc, 1∶200) Rac1 (Santa Cruz, 1∶200) and GAPDH (Sigma-aldrich, 1∶4000). Proteins were detected using enhanced chemiluminescence (ECL) with horseradish peroxidase conjugated appropriate secondary antibodies (Amersham).

### NFκB Activity Assay

The NFκB activities in HMECs were investigated by luciferase activities assay. HMECs (100, 000 cells/well) were seeded in six-well plates the day before transfection. Transfection was performed using Lipofectamine2000. Each well contained 200 ng of either empty pGL3 (Promega, New South Wales, Australia) or NFκB-driven luciferase (Stratagene, La Jolla, CA) pGL3 vector and 50 ng pRL-SV40 (Promega) mixed with 0.5 µl of Lipofectamine2000 in the presence of 250 µl of Opti-MEM. Cells were transfected for 5 h and then incubated with complete medium for 24 h. After 24 h cells were treated with TNFα in the absence or presence of Ac2-26 peptide or DPI for 24h. NFκB-driven luciferase activity in cells was determined using a Dual-Luciferase reporter assay system (Promega, New South Wales, Australia) and measured on a Polarstar microplate reader. Transfection efficiency was normalized with the Renilla luciferase containing plasmid pRL-SV40 according to manufacturer’s instructions.

### Data and Statistics

Data are presented as mean ± standard error of the mean (SEM). The mean data were analyzed with one-way analysis of variance (one-way ANOVA) followed by Newman-Keuls post hoc or *t*-test (for multiple comparisons). A *P* value of less than 0.05 was regarded as statistically significant.

## Results

### Effects of TNFα on Superoxide Generation, ICAM-1 and VCAM-1 Expression in HMECs

**Figure 1:**
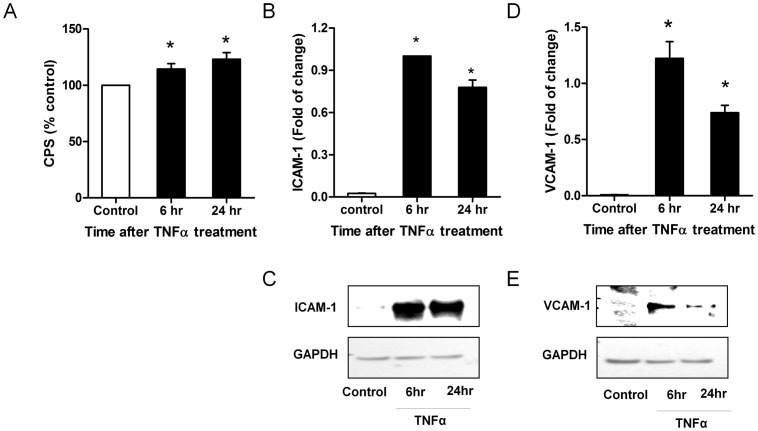
Tumour necrosis factor (TNFα) stimulated NADPH-dependent superoxide release and cell adhesion molecule expression in HMECs. (A) Superoxide production detected by lucigenin enhanced chemiluminescence (normalized to control without TNFα stimulation) following 6 and 24 h TNFα treatment (20 ng/ml). (B) Intracellular cell adhesion molecule-1 (ICAM-1) mRNA (expressed as fold change) following 6 and 24 h TNFα stimulation (C) ICAM-1 protein expression following 6 and 24 h TNFα stimulation. (D) Vascular cell adhesion molecule-1 (VCAM-1) mRNA (expressed as fold change) following 6 and 24 h TNFα stimulation (E) VCAM-1 protein expression following 6 and 24 h TNFα stimulation. Data are mean ± SEM, *n* = 5 to 8. * *P*<0.05 *vs* control.

Treatment of HMECs with TNFα (20 ng/ml) stimulated NADPH-dependent lucigenin enhanced chemiluminescence (Figure 1A). At 6 h, chemiluminescence was increased to 114±4.7% of control (*n* = 6, *P*<0.05, one way ANOVA) and increased to 123.2±5.8% of control (*n* = 5, *P*<0.01, one way ANOVA) after 24 h. TNFα is a well known pro-inflammatory cytokine and we [29] and others [13], [23], have previously shown that TNFα induces gene expression of adhesion molecule in endothelial cells. Similar to its stimulatory effect on NADPH-dependent superoxide anion generation, TNFα induced both gene and protein expression of ICAM-1 (Figure 1B and C) and VCAM-1 (Figure 1D and E) expression at 6 h and 24 h.

### The Stimulatory Effect of TNFα is NADPH Oxidase Dependent

**Figure 2:**
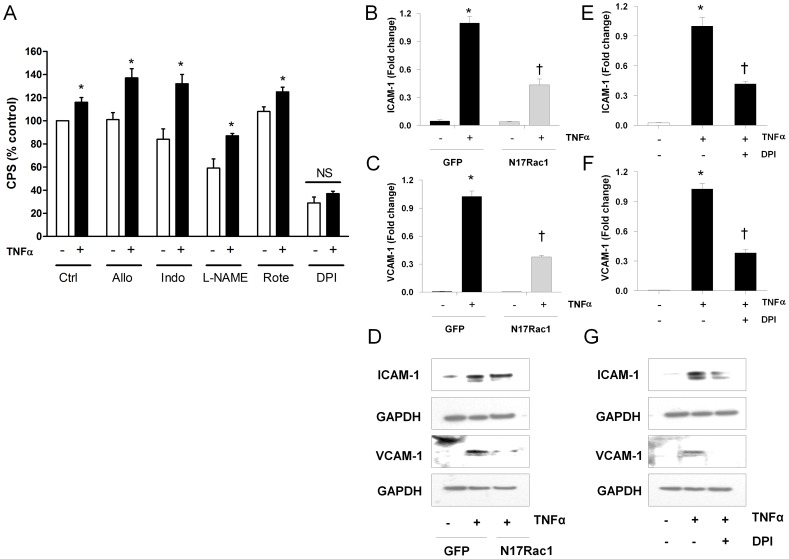
The stimulatory effect of tumour necrosis factor (TNFα) is NADPH oxidase dependent in HMECs. (A) Inhibitors of other enzymatic sources of ROS, allopurinol (Allo, 100 µM), indomethacin (Indo, 3 µM), L-NAME (100 µM) and rotenone (Rote, 1 µM) did not affect TNFα-induced superoxide generation, but it was inhibited by the Nox inhibitor diphenyleneiodonium (DPI, 1 µM). Cells were treated with TNFα (20 ng/ml) for 24 h prior to incubation with inhibitors, which were incubated with HMECs for 30 min prior to measurement of superoxide. (B, C) TNFα (20 ng/ml) stimulated ICAM-1 and VCAM-1 mRNA upregulation in cells transfected with control GFP plasmid. Dominant negative Rac1 (N17Rac1) reduced TNFα-mediated ICAM-1and VCAM-1 mRNA upregulation. mRNA expression was normalized to control with TNFα stimulation in cells transfected with GFP. (D) TNFα (20 ng/ml) stimulated ICAM-1 and VCAM-1 mRNA upregulation in cells transfected with control GFP plasmid. Dominant negative Rac1 (N17Rac1) reduced TNFα-mediated ICAM-1and VCAM-1 protein expression. GAPDH was used as a reference. (E,F) DPI (1 µM) suppressed the stimulatory effect of TNFα (20 ng/ml) on mRNA expression of ICAM-1 and VCAM-1 respectively. mRNA expression was expressed as fold change and normalized to control (Ctrl) with TNFα stimulation. (G) DPI (1 µM) suppressed the stimulatory effect of TNFα (20 ng/ml) on protein expression of ICAM-1 and VCAM-1. GAPDH was used to confirm equal loading. Data are mean ± SEM*; n* = 3. * *P*<0.05 *vs* control without TNFα stimulation; † *P*<0.05 *vs* control with TNFα stimulation.

To investigate which enzyme sources are involved in the TNFα-stimulated increase in superoxide production, cells were treated with pharmacological inhibitors of these enzymes 30 min prior to superoxide measurement (Figure 2A). Whilst a number of the inhibitors altered basal superoxide generation (i.e. superoxide generated in the absence of TNFα), inhibition of xanthine oxidase (allopurinol, 100 µM), cyclooxygenase (indomethacin, 3 µM), endothelial nitric oxide synthase (L-NAME, 100 µM) as well as mitochondrial sources (rotenone, 1 µM) failed to inhibit the TNFα-stimulated increase in superoxide generation. Only inhibition of NADPH oxidase with DPI (1 µM) abrogated the TNFα stimulated increase in superoxide generation, suggesting that NADPH oxidase is a major source of TNFα-stimulated superoxide release in these cells. We next explored whether interfering with the assembly of a functional NADPH oxidase affects TNFα mediated ICAM-1 and VCAM-1expression by transfecting HMECs cells with a plasmid carrying dominant negative Rac1 (N17Rac1). Endothelial cells transfected with N17Rac1 plasmid showed a marked increase in N17Rac1 protein compared to cells transfected with the control GFP plasmid (Figure S3 D), indicating an efficient transfection of endothelial cells with N17Rac1. Interestingly, N17Rac1 transfected endothelial cells showed a marked reduction in the TNFα-induced upregulation of ICAM-1 (Figure 2B and D) and VCAM-1 (Figure 2C and D). Similarly, DPI (1 µM) significantly decreased the TNFα-induced upregulation of both ICAM-1(Figure 2E and G) and VCAM-1 (Figure 2F and G), confirming that NADPH oxidase-derived ROS are involved in TNFα-stimulated expression of cell adhesion molecules.

### Effects of Ac2-26 on Superoxide Generation and ICAM-1 and VCAM-1 Expression

**Figure 3:**
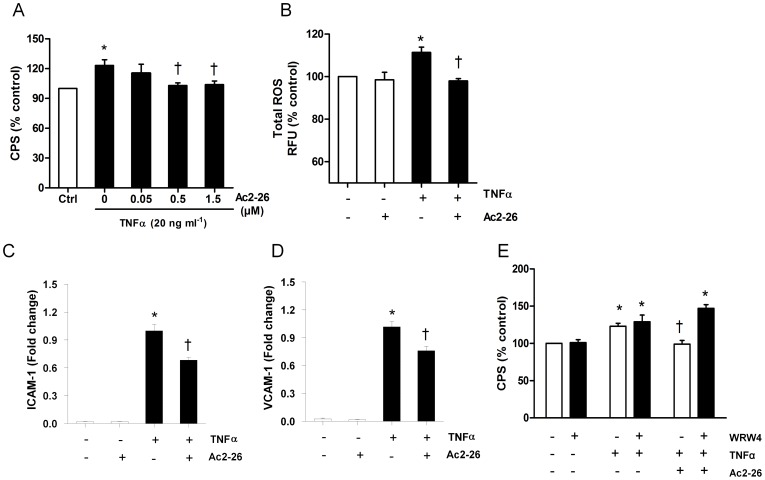
Annexin-1 peptide Ac2-26 inhibited tumour necrosis factor (TNFα)-mediated response in HMECs. Ac2-26 (0.5 and 1.5 µM) suppressed TNFα (20 ng/ml) stimulated (A) NADPH dependent superoxide production measured by lucigenin-enhanced chemiluminescence and (B) total intracellcular ROS production detected by DCFH_2_-DA. (C) Ac2-26 (0.5 µM) inhibited the stimulatory effect of TNFα on ICAM-1 mRNA expression, without affecting its basal level. (D) Ac2-26 (0.5 µM) inhibited the stimulatory effect of TNFα on VCAM-1 mRNA expression, without affecting its basal level. (E) FPRL-1 antagonist WRW4 prevented the inhibitory effect of Ac2-26 on TNFα (20 ng/ml) stimulated superoxide generation. mRNA expression data was normalized to control with TNFα stimulation. Data are mean ± SEM, *n* = 3 to 5. * *P*<0.05 *vs* control without TNFα stimulation; ^†^ *P*<0.05 *vs* control with TNFα.

We next examined the effect of the annexin peptide Ac2-26 on both basal and TNFα stimulated superoxide release, ICAM-1and VCAM-1 gene expression since Ac2-26 has previously been shown to block the response to TNFα. At concentrations below 1.5 µM basal superoxide release was unchanged (Figure S1A). Pretreatment of cells with Ac2-26 30 min prior to TNFα stimulation (20 ng/ml) dose-dependently reduced superoxide generation (Figure 3A). Ac2-26 (0.5 µM) significantly reduced TNFα-stimulated superoxide release from 123±6% to 103±3% of control (*n* = 5, *P*<0.05 *vs* TNFα alone). For further studies an Ac2-26 concentration of 0.5 µM was used since this reduced TNFα-stimulated superoxide release without affecting basal release. To validate the inhibitory effect of Ac2-26 on ROS generation, we also assayed total ROS production using DCFH_2_-DA fluorescence and found that Ac2-26 completely abolished the TNFα-induced intracellular ROS generation in endothelial cells (Figure 3B). Moreover, TNFα-induced gene expression of ICAM-1 (Figure 3C) and VCAM-1 (Figure 3D) was also suppressed by Ac2-26, demonstrating its anti-inflammatory activity upon TNFα stimulation without exerting basal effects.

### The Anti-inflammatory Effect of Ac2-26 is FPRL-1 Receptor Specific

To confirm that the inhibitory effect of Ac2-26 is specific, we used the FPRL-1 antagonist WRW4. HMECs were treated with WRW4 (10 µM) for 30 minutes prior to Ac2-26 and TNFα treatment. WRW4 did not alter basal or TNFα-stimulated superoxide production (Figure 3E) but abrogated the Ac2-26 inhibition of TNFα-induced ROS production (Figure 3E). This pattern was also observed for basal and TNFα-induced ICAM-1 gene upregulation (Figure S2). These results indicate that Ac2-26 acts via FPRL-1 receptor to block the TNFα responses.

### TNFα Induced ICAM-1 and VCAM-1 Expression Requires Rac-1 Dependent NADPH Oxidase Activation

**Figure 4:**
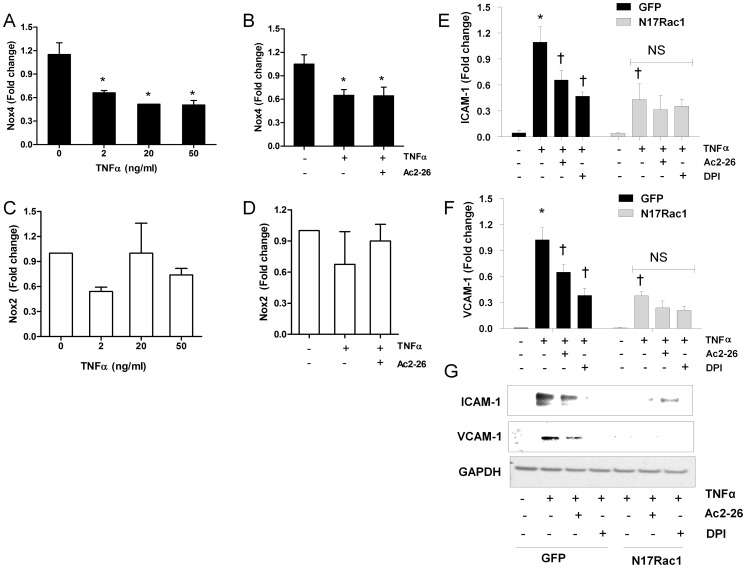
The stimulatory effect of tumour necrosis factor (TNFα) on adhesion molecule gene upregulation requires Rac1 in HMECs. (A) TNFα (2–50 ng/ml) reduced Nox4 gene expression (B) and pretreatment of Ac2-26 (0.5 µM) did not affect the TNFα mediated mRNA downregulation. TNFα (2–50 ng/ml) alone (C) and in combination of Ac2-26 (0.5 µM) (D) did not affect Nox2 mRNA expression. TNFα (20 ng/ml) stimulated ICAM-1 mRNA (E) and protein (G) and VCAM-1 mRNA (F) and protein (G) upregulation was blunted by Ac2-26 (0.5 µM) and diphenyleneiodonium (DPI, 1 µM) in cells trasnfected with control GFP plasmid. Dominant negative Rac1 (N17Rac1) reduced TNFα-mediated ICAM-1 and VCAM-1 upregulation and this inhibition was not potentiated by pretreatment of Ac2-26 and DPI. mRNA expression was normalized to control with TNFα stimulation in cells transfected with GFP. GAPDH was used to confirm equal loading. Data are mean ± SEM, *n* = 5 to 8. * *P*<0.05 *vs* control without TNFα stimulation; ^†^ *P*<0.05 *vs* control with TNFα stimulation. There was no difference (not significant, NS) between control Ac2-26 and DPI in the presence of TNFα in cells transfected with N17Rac1.

Having shown that Ac2-26 actions are NADPH oxidase dependent, we examined whether this effect was due to alterations in NADPH oxidase subunit expression. We measured the gene expression of Nox2 and Nox4 catalytic subunits following 24 h TNFα stimulation in HMECs. Surprisingly TNFα (2-50 ng/ml) suppressed Nox4 gene expression (Figure 4A), suggesting Nox4 was not involved in TNFα-mediated superoxide release. Co-treatment of Ac2-26 did not modify the inhibitory effect of TNFα on Nox4 gene expression. Neither did TNFα alone nor in combination with Ac2-26 affect the gene expression of Nox2 (Figures 4C and 4D).Similarly, TNFα alone or in combination with Ac2-26 did not affect gene expression of associated subunits: p22phox, p67phox (Figures S3A and S3B) and protein expression of Rac1 (Figures S3C and S3D). This suggests that TNFα does not regulate the gene expression of Nox2 or other important subunits in endothelial cells.

Since we were unable to detect any change in gene expression (Nox2, p22phox and p67phox), we speculated that Ac2-26 might interfere with the assembly of NADPH oxidase thereby suppressing TNFα mediated ICAM-1 and VCAM-1 gene expression. HMECs were therefore transfected with plasmid carrying a dominant negative Rac1 (N17Rac1) before addition of either TNFα or Ac2-26. In cells transfected with a control GFP plasmid, TNFα stimulated expression of ICAM-1(Figure 4E and G) and VCAM-1 (Figure 4F and G) was abrogated by Ac2-26 and the Nox inhibitor DPI as expected. Interestingly, N17Rac1 transfected endothelial cells showed a marked reduction in the TNFα mediated upregulation of ICAM-1 and VCAM-1 expression as shown previously (Figure 2). Moreover the inhibitory effect of Ac2-26 and DPI on TNFα-induced expression of ICAM-1 (Figure 4E and G) and VCAM-1 (Figure 4F and G) was no longer observed in N17Rac1 transfected cells. These findings suggest that TNFα activates Rac1 dependent NADPH oxidase activity to increase expression of ICAM-1 and VCAM-1, and this enzyme assembly process is suppressed by Ac2-26. Furthermore, we found that pretreatment of Ac2-26 for 0.5 h also reduced the PMA stimulated superoxide generation in phagocytic cells (DMSO differentiated HL-60 cells [29] (Figure S4). Overall our findings suggest that Ac2-26 prevents TNFα mediated NADPH oxidase activation to suppress both ICAM-1 and VCAM-1 expression.

### Ac2-26 Antagonised TNFα Induced NF-κB Promoter Activity

**Figure 5:**
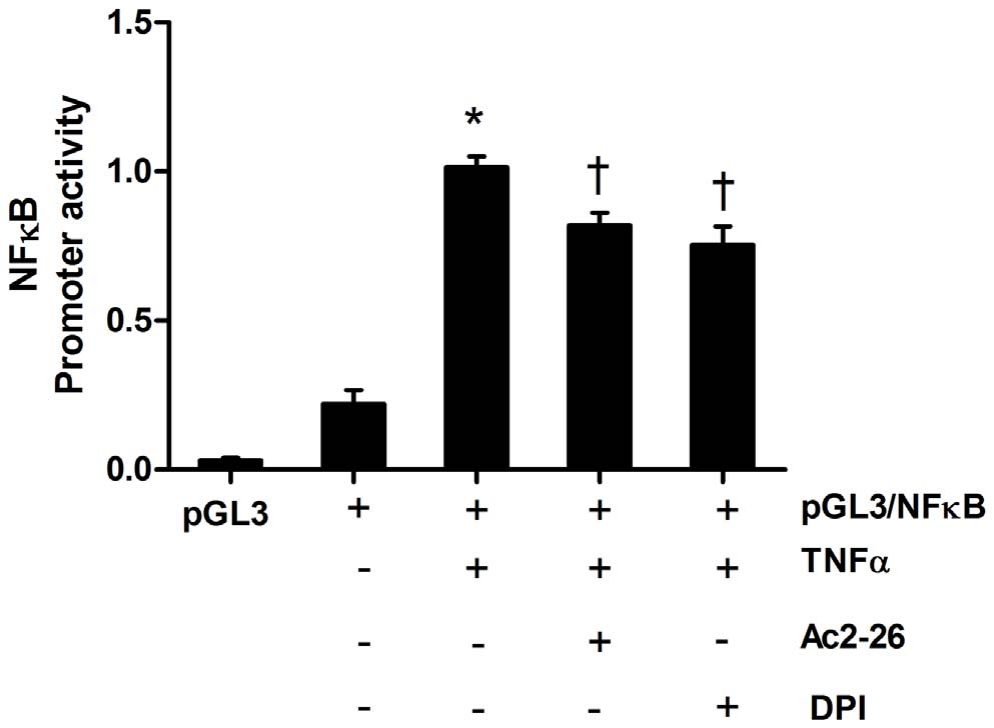
The effect of annexin-1 peptide Ac2-26 on TNFα-induced NF-κB promoter activity in HMECs. TNFα (20 ng/ml) induced NF-κB promoter activity is decreased by pretreatment of Ac2-26 (0.5 µM) and diphenyleneiodonium (DPI, 1 µM). Luciferase activity is expressed as relative luminescence units (RLU) and is normalised to control without TNFα stimulation. Data are mean ± SEM, *n* = 4. *P<0.05 vs control without TNFα, ^†^P<0.05 vs control with TNFα.

To further elucidate the effect of Ac2-26 on TNFα downstream signaling process, we transfected endothelial cells with human nuclear factor kappa B(NF-κB) promoter cloned in pGL3 luciferase reporter vector (pGK3/NF-κB) and tested the effects of TNFα alone and in combination with Ac2-26. As expected, TNFα significantly increased the NF-κB promoter activity (Figure 5) and this was reduced to the same extent by Ac2-26 and DPI.

## Discussion

Annexin 1 and its derived N-terminus peptide Ac2-26 are well known to exert anti-inflammatory activities in inflammatory cells such as neutrophils and macrophages [30], [31], [32]. Information about their actions in other cells that are important to inflammation particularly endothelial cells is limited [33]. The present study addressed this aspect in human endothelial cells, focusing on the effect of Ac2-26 on ROS generation, ICAM-1and VCAM-1 induction following stimulation with the proinflammatory cytokine TNFα. This is the first study to illustrate that Ac2-26 inhibits the activation of Rac1-depedent NADPH oxidase to suppress stimulated superoxide generation in endothelial cells, leading to downregulation of both ICAM-1 and VCAM-1 expression.

We and others have previously shown that TNFα-mediated responses such as superoxide generation and adhesion molecule expression are NADPH oxidase-dependent in human endothelial cells [2], [16], [17], [29]. We confirmed this by demonstrating that TNFα mediated superoxide release was sensitive to DPI and the induced ICAM-1and VCAM-1 gene upregulation were blocked by inhibition of NADPH oxidase with the use of dominant negative Rac1 (N17Rac1) and DPI. Several studies have shown that Rac1 is required for the assembly of an active NADPH oxidase in human endothelial cells [16], [17]. Chen et al. [16], [19] recently reported that Rac1 and an NADPH oxidase-dependent pathway was responsible for the TNFα-mediated responses including increases in ICAM-1, VCAM-1 and MCP-1 expression. Furthermore, Li et al. showed that TNFα binds to its adaptor proteins, TNF receptor associated factors (TRAF4), to promote the membrane association of p47phox in HMECs [17]. Similarly endothelial cells isolated from either p47phox or Nox2 knockout animals also showed a reduction in TNFα-induced ICAM-1 gene expression [2], [17]. Likewise, subcutaneous infusion of a specific Nox2 inhibitor peptide gp91ds-tat reduced angiotensin II-induced ICAM-1 protein expression in the rat aortic endothelium [34]. Clearly, TNFα activates the assembly of a Nox2-based NADPH oxidase to promote superoxide generation leading to an increase in ICAM-1 gene expression.

TNFα has also been shown to modify the gene expression of catalytic subunits such as Nox2 in several cell types. We have previously demonstrated that TNFα increases Nox2 gene expression in HEK293 cells [35]. Transgenic mice with an overexpression of TNFα in endothelial cells also exhibited an increase in Nox2 mRNA expression [36]. Similarly, Nox2 mRNA in monocytic and microglial cell lines was upregulated following lipopolysaccharide/interferon gamma treatment via the NF-κB signaling pathway [37]. In contrast, here we could not find any effect of TNFα on the gene expression of Nox2 and the associated subunits p22phox and p67phox. Nox4 is another important Nox subtype found in human endothelial cells and its superoxide generating capacity is independent of Rac1. Surprisingly we found TNFα suppressed Nox4 mRNA in the present study whilst others showed that TNFα elevated Nox4 gene expression in porcine cerebral endothelial cells [38]. The difference in TNFα mediated gene alteration between these cells [38] may relate to the specific subtype of protein kinase C second messenger that is activated upon TNFα stimulation, and remains to be clarified. For example, Frey et al [39] previously showed that protein kinase Cζ regulates TNFα induced NADPH oxidase activation and this involves phosphorylation of the associated subunits p47phox or p67phox [40]. On the other hand, Xu et al. [41] found that PKCε downregulates Nox4 and PKCα upregulates Nox4, suggesting an interrelated regulatory mechanism between protein kinase C subtypes and Nox4 gene regulation following TNFα stimulation. Nevertheless, our findings suggest that TNFα-induces Nox2 based NADPH oxidase activity rather than the expression of either Nox2 or Nox4 gene expression to stimulate ROS generation in HMECs. This is well supported by a recent study which showed that TNFα treatment stimulated total ROS production in HEK cells overexpressing Nox2 but not Nox4 [42]. The other potential candidate for superoxide generation in human endothelial cells is Nox5 [24] and its activation relies solely on calcium [43]. Furthermore Montezano et al. [44] demonstrated that Nox5 activation was independent of Rac following angiotensin II stimulation in HMECs. Therefore Nox5 is unlikely to contribute to TNFα induced superoxide release in the present study.

TNFα-induced NADPH oxidase derived ROS have been shown to regulate downstream signaling by mitogen-activated protein (MAP) kinases including p38 MAPK, extracellular signal-regulated kinase (Erk1/2) and c-Jun terminal kinases (JNK) in endothelial cells [17], [20]. Li et al. demonstrated that the binding of TRAF4 to p47phox promoted the phosphorylation of p38 MAPK and Erk1/2 but not JNK [17]. On the other hand, Lin et al. reported that inhibition of JNK or p38 MAPK but not of Erk suppressed NADPH oxidase dependent-TNFα mediated ICAM-1 and VCAM-1 gene upregulation [20]. Although these findings are inconsistent, they both support a role for NADPH oxidase-derived ROS in the regulation of MAPK phosphorylation. It has been established that MAPK phosphorylation activates the transcription factor NF-κB to promote cell adhesion molecule gene expression in endothelial cells [45]. Consistent with the findings of Yin et al. [45], we demonstrated that TNFα enhanced the promoter activity of NF-κB. Min et al. recently demonstrated that TNF-related activation-induced cytokine (TRANCE) or receptor activator of NF-κB ligand increases ICAM-1 protein and NF-κB DNA binding in human endothelial cells and both responses are abrogated by inhibition of protein kinase C, phospholipase and antioxidant [46]. Phospholipase and protein kinase C have previously been shown to regulate NADPH oxidase activation [14], so TNFα activation of the Rac1 pathway could involve phospholipase and this aspect warrants further investigation.

Endothelial cells play an important role in inflammation since they serve as the primary transendothelial migration points for blood borne leucocytes to reach inflamed tissues [47]. Accumulating evidence has implicated a role for NADPH oxidase-derived ROS in endothelial activation during the inflammatory response [2], [12], [16], [17], [48]. Limited studies conducted by us [4] and others [33] have shown a protective action of annexin peptide Ac2-26 in cell types other than inflammatory cells such as cardiac myocytes and endothelial cells. Using human endothelial cells, we confirmed the inhibitory effect of Ac2-26 on superoxide generation and ICAM-1 gene upregulation was indeed FPRL-1 receptor specific. We further demonstrated a novel inhibitory mechanism exerted by Ac2-26 on superoxide generation by showing Ac2-26 prevents TNFα mediated assembly of Rac1-dependent NADPH oxidase, leading to a downregulation of NF-κB-induced ICAM-1 and VCAM-1expression. How Ac2-26 interferes with the activation of NADPH oxidase is not fully understood. Annexin-1 or Ac2-26 peptide has previously been shown to inhibit phospholipase A2 [49], [50] and phosopholipases are involved in the activation of NADPH oxidase in several cell types [14], [51]. We therefore propose that Ac2-26 may target phospholipase and this then interferes with NADPH oxidase assembly to reduce superoxide generation.

Interestingly, endogenous annexin has been detected in endothelial cells following an inflammatory insult [52], suggesting that spontaneous local production of annexin may well be a natural cell defense mechanism for the endothelial cells. However, elevating the production of annexin might not always be favorable. Williams et al. recently showed that cleaved C-terminal annexin peptide increased the clustering of ICAM-1 proteins around neutrophils, facilitating their transmigration across endothelial cells, whilst Ac2-26 (annexin derived peptide with N-terminal) has the opposite effect [53]. Furthermore, we demonstrated that Ac2-26 inhibited Rac1-dependent NADPH oxidase-derived superoxide generation in both phagocytes and endothelial cells. However, Ac2-26 attenuated (inhibits ∼25%) phagocytic NADPH oxidase (mainly Nox2) at 5 µM, whereas 0.5 to 1.5 µM concentration of Ac2-26 completely abolished NADPH oxidase activity in endothelial cells. This finding is consistent with a previous study by Karlsson et al. that 20 to 100 µM of Ac2-26 is required to block neurophil NADPH oxidase activity induced by formylated peptide N-Formyl-Met-Leu-Phe (fMLP) [54]. This may be due to the higher expression level of NADPH oxidase subunits and activities in phagocytic as compared to endothelial cells. Indeed, earlier studies have shown that mRNA copy number of Nox2 is many fold higher in phagocytic cells than endothelial cells [23]. Thus, sub-micromolar levels of Ac2-26 are sufficient to block endothelial NADPH oxidase activity.

We and others have previously shown that both phagocytic and vascular NADPH oxidases are important in inflammatory processes such as extravasation and transmigration of inflammatory cells and activation of endothelial cells, including increases in cell adhesion molecule expression and endothelial dysfunction, which are important sources for the initiation and progression of reperfusion injury and artery diseases such as atherosclerosis [2],[48],[55],[56]. Therefore, exogenous application of Ac2-26 could have therapeutic potential stemming from suppression of ROS generation derived from both phagocytic and vascular NADPH oxidases.

In conclusion, our findings suggest that Rac1-dependent superoxide generation is essential for TNFα-mediated upregulation of ICAM-1 and VCAM-1 in endothelial cells. This is the first study to demonstrate that Ac2-26 inhibits superoxide generation in endothelial cells by interfering with the assembly of NADPH oxidase.

## Supporting Information

Figure S1 **The effects of annexin-1 peptide Ac2-26 on superoxide generation.** Ac2-26 did not affect superoxide generation detected by lucigenin-enhanced chemiluminescence.
(TIF)

Figure S2 **The effect of annexin peptide Ac2-26 via FRL-1on ICAM-1 gene expression.** FPRL-1 antagonist WRW4 inhibited the effect of Ac2-26 (0.5 µM) by restoring the TNFα stimulated mRNA expression of ICAM-1 in the presence of Ac2-26. Cells were pretreated 30 min with WRW4(10 µM) then incubated with Ac2-26 alone (0.5 µM) or TNFα (20 ng/ml)+Ac2-26 for 6h. TNFα was added 30 min after Ac2-26. mRNA expression was normalized to control with TNFα stimulation. Data are mean ± SEM, *n* = 3 to 5. * *P*<0.05 *vs* control without TNFα stimulation; ^†^ *P*<0.05 *vs* control with TNFα.
(TIF)

Figure S3 **The effects of annexin-1 peptide Ac2-26 on Nox subunit and Rac1 expression in HMECs.** TNFα (2-50 ng/ml), alone (A) and in combination with Ac2-26 (B) did not affect mRNA expression of p22phox and p67phox. (C) TNFα treatment for 6 or 24 h did not alter Rac1 protein expression. (D) TNFα and in combination with either Ac2-26 or DPI did not affect Rac1 proteins expression. mRNA expression data was normalized to control (Ctrl) without TNFα. Data are mean ± SEM, *n* = 4.
(TIF)

Figure S4 **The effects of annexin-1 peptide Ac2-26 on superoxide generation in DMSO differentiated HL-60 cells.** PMA stimulated the superoxide generation detected by lucigenin-enhanced chemiluminescence in DMSO differentiated HL-60 cells and this is reduced by pretreatment with Ac2-26. Data are mean ± SEM, *n* = 8 to 9. * *P*<0.05 vs control (Ctrl) without PMA, ^†^P<0.05 vs control with PMA.
(TIF)
