# Supplementary figures and images for: Annexin Peptide Ac2-26 Suppresses TNFα-Induced Inflammatory Responses via Inhibition of Rac1-Dependent NADPH Oxidase in Human Endothelial Cells
Source: PLoS One. 2013 Apr 24;8(4):e60790. doi: 10.1371/journal.pone.0060790 (PMC3634803; doi:10.1371/journal.pone.0060790)

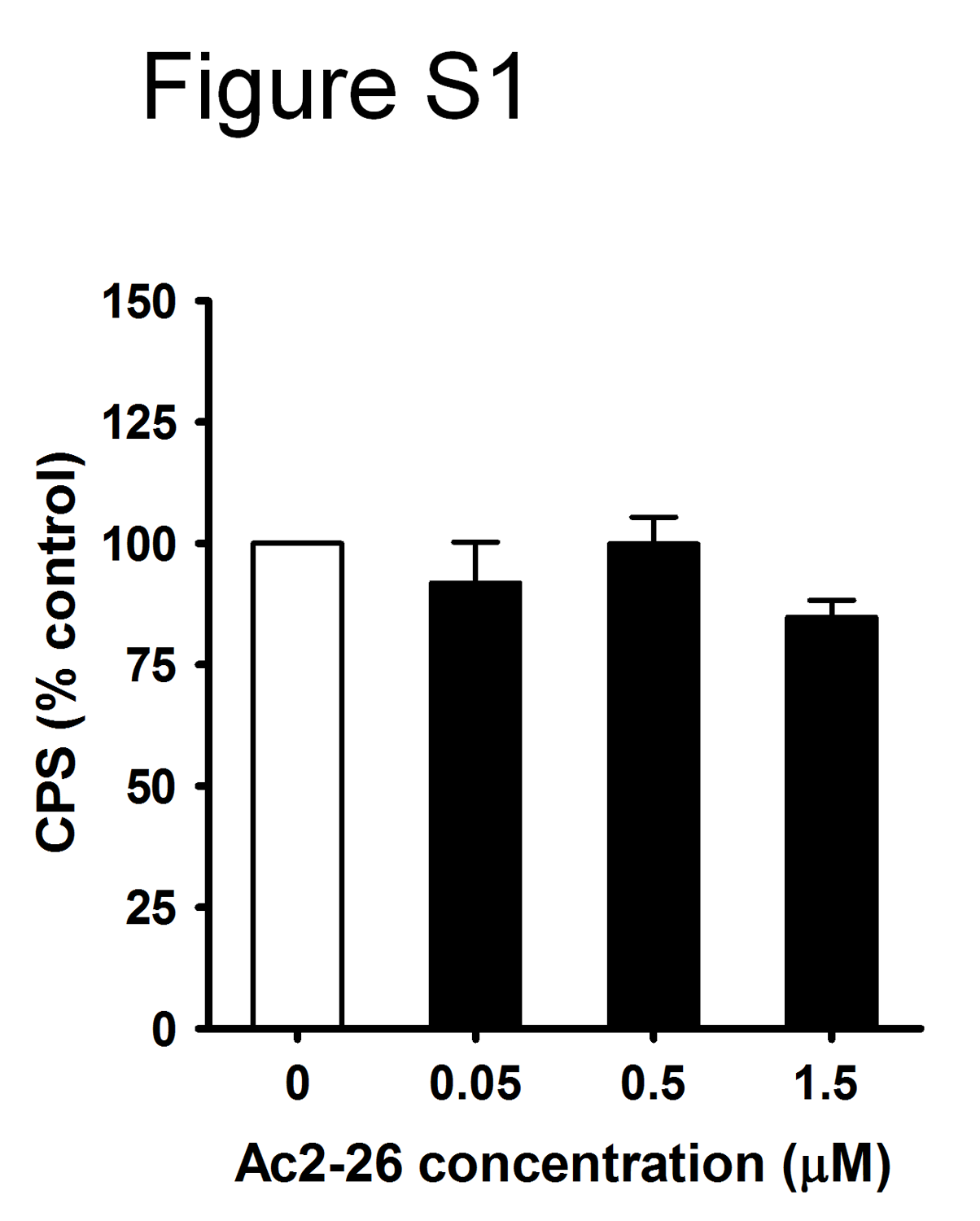

Supplement: Figure S1 — The effects of annexin-1 peptide Ac2-26 on superoxide generation. Ac2-26 did not affect superoxide generation detected by lucigenin-enhanced chemiluminescence. (TIF) [file pone.0060790.s001.tif]

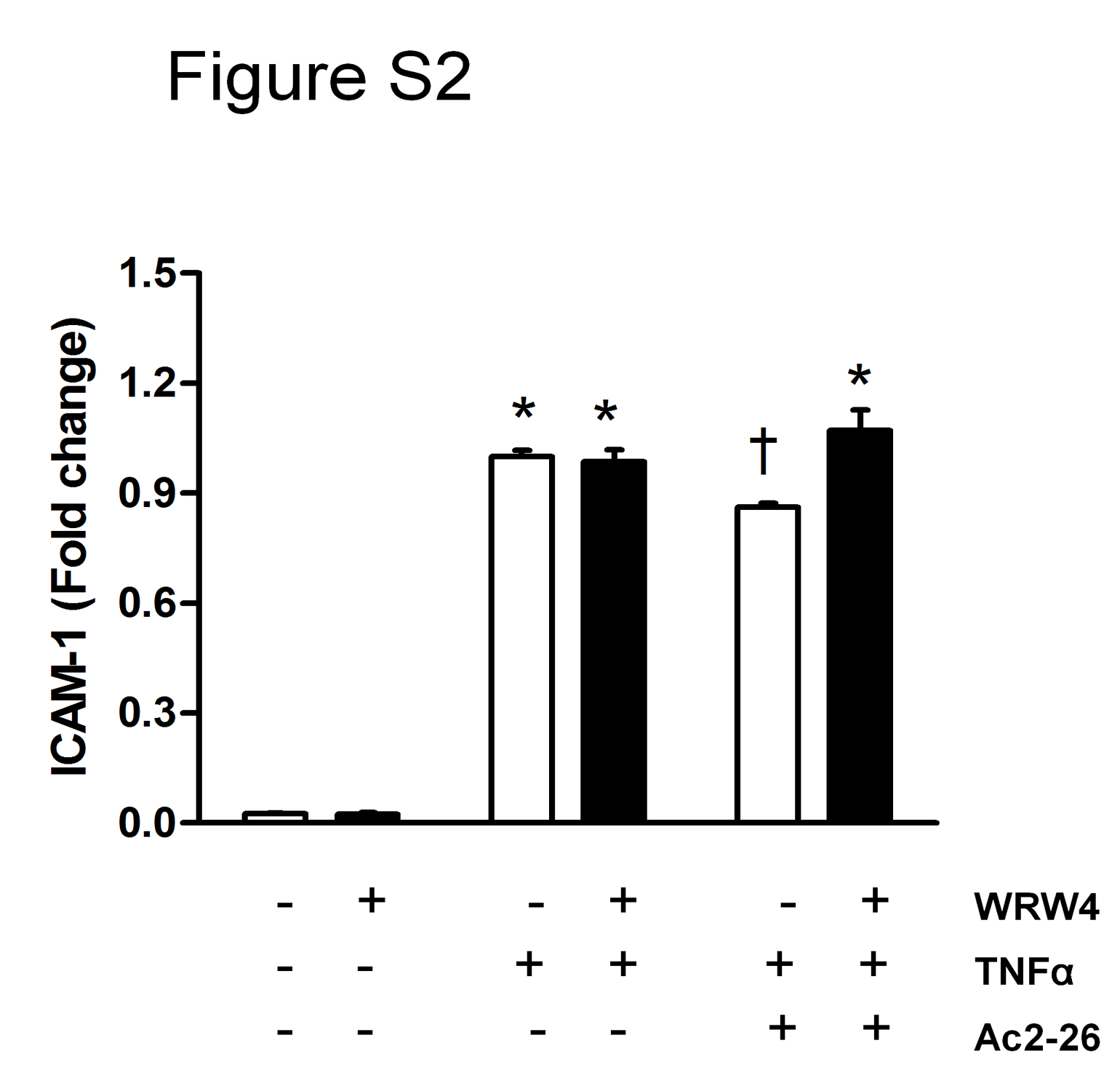

Supplement: Figure S2 — The effect of annexin peptide Ac2-26 via FRL-1on ICAM-1 gene expression. FPRL-1 antagonist WRW4 inhibited the effect of Ac2-26 (0.5 µM) by restoring the TNFα stimulated mRNA expression of ICAM-1 in the presence of Ac2-26. Cells were pretreated 30 min with WRW4(10 µM) then incubated with Ac2-26 alone (0.5 µM) or TNFα (20 ng/ml)+Ac2-26 for 6h. TNFα was added 30 min after Ac2-26. mRNA expression was normalized to control with TNFα stimulation. Data are mean ± SEM, n = 3 to 5. * P<0.05 vs control without TNFα stimulation; † P<0.05 vs control with TNFα. (TIF) [file pone.0060790.s002.tif]

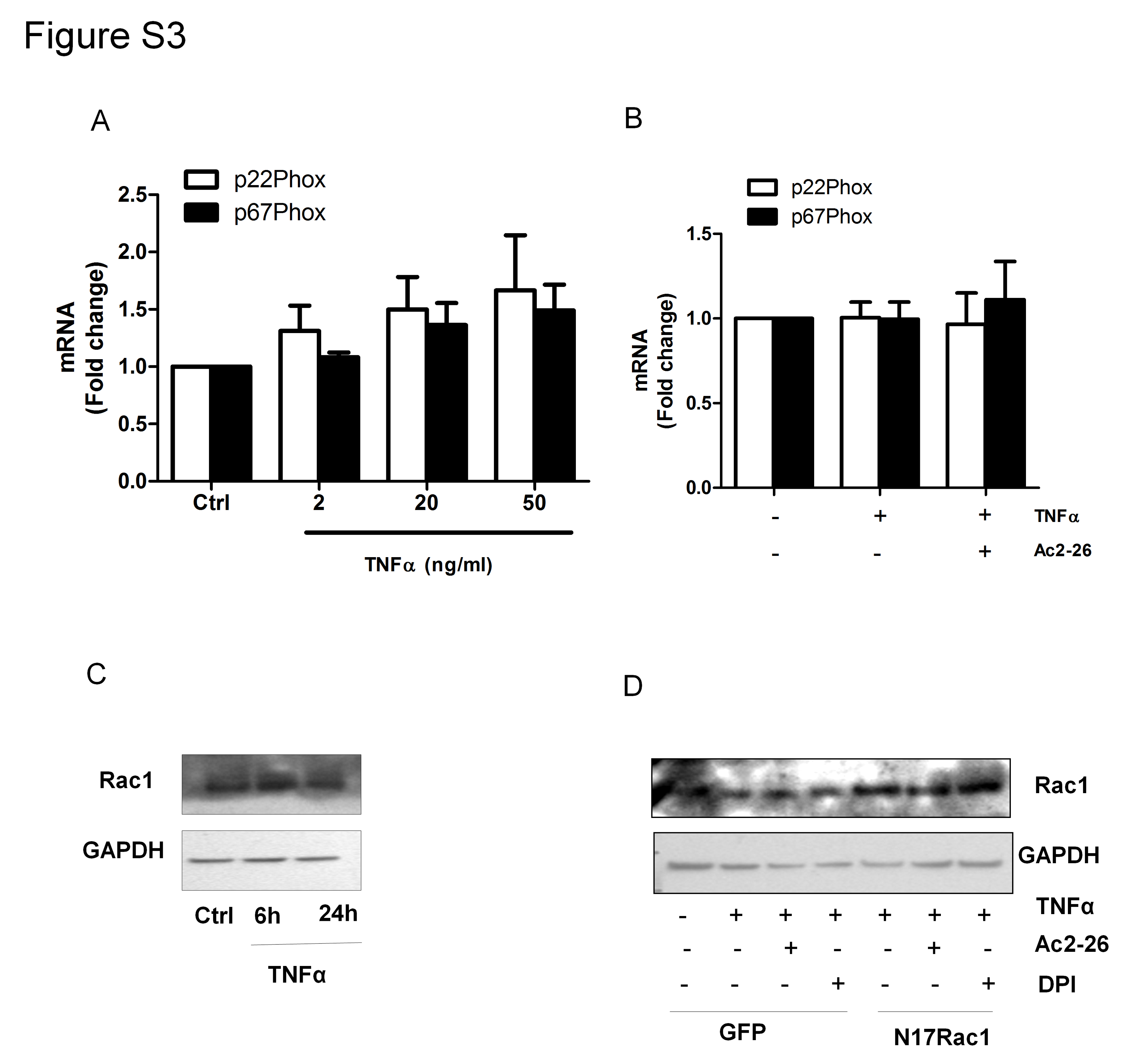

Supplement: Figure S3 — The effects of annexin-1 peptide Ac2-26 on Nox subunit and Rac1 expression in HMECs. TNFα (2-50 ng/ml), alone (A) and in combination with Ac2-26 (B) did not affect mRNA expression of p22phox and p67phox. (C) TNFα treatment for 6 or 24 h did not alter Rac1 protein expression. (D) TNFα and in combination with either Ac2-26 or DPI did not affect Rac1 proteins expression. mRNA expression data was normalized to control (Ctrl) without TNFα. Data are mean ± SEM, n = 4. (TIF) [file pone.0060790.s003.tif]

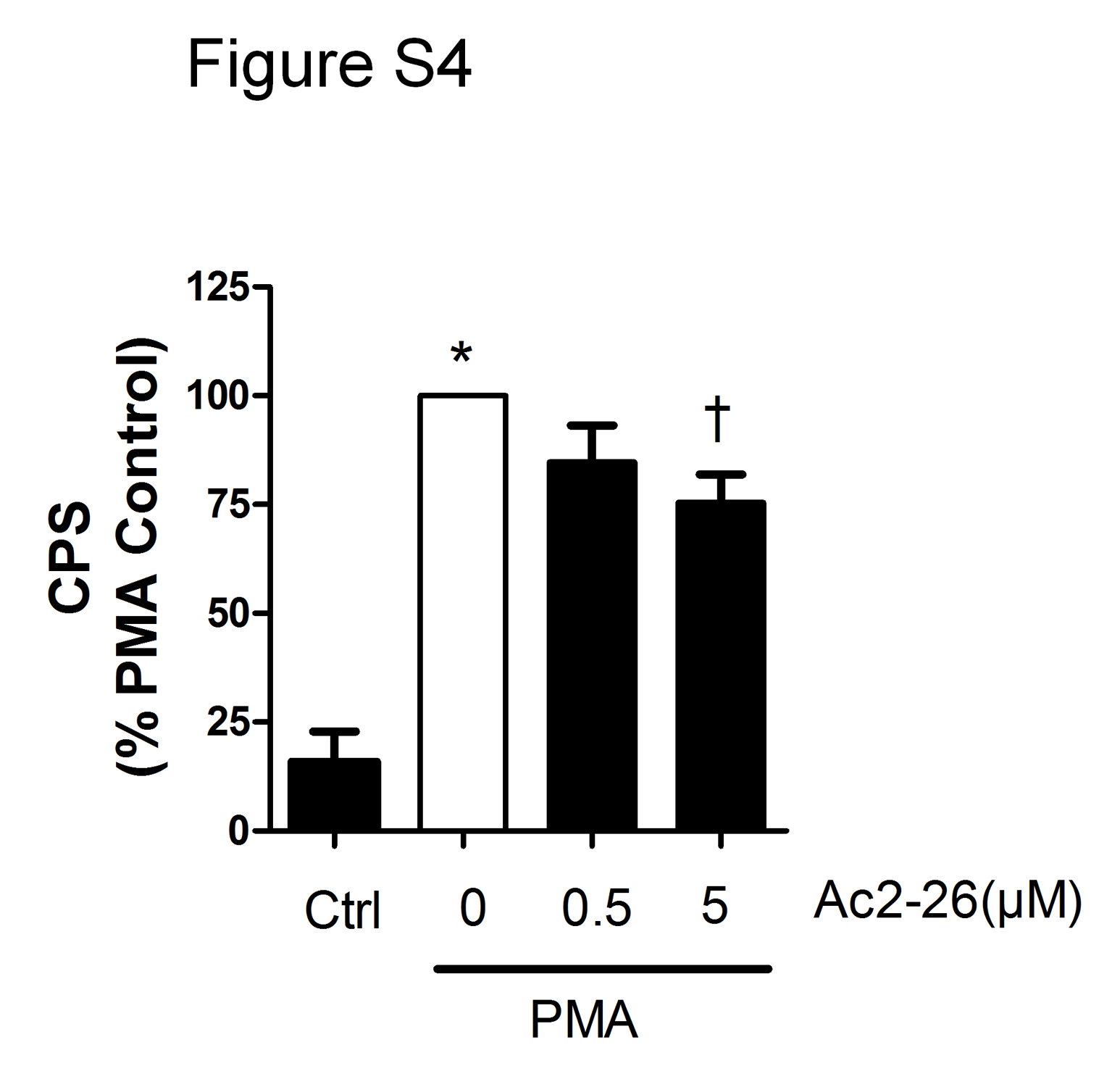

Supplement: Figure S4 — The effects of annexin-1 peptide Ac2-26 on superoxide generation in DMSO differentiated HL-60 cells. PMA stimulated the superoxide generation detected by lucigenin-enhanced chemiluminescence in DMSO differentiated HL-60 cells and this is reduced by pretreatment with Ac2-26. Data are mean ± SEM, n = 8 to 9. * P<0.05 vs control (Ctrl) without PMA, †P<0.05 vs control with PMA. (TIF) [file pone.0060790.s004.tif]
